# Expression of ADRB2 in children with neuroblastoma and its influence on prognosis

**DOI:** 10.3389/fsurg.2022.1026156

**Published:** 2022-11-02

**Authors:** Lijian Cao, Qingqing Liu, Yue Ma, Fengling Shao, Zhenzhen Zhao, Xiaobin Deng, Jianwu Zhou, Shan Wang

**Affiliations:** Department of Pediatric Surgical Oncology, Children’s Hospital of Chongqing Medical University; and the National Clinical Research Center for Child Health and Disorders, Ministry of Education Key Laboratory of Child Development and Disorders, Chongqing Key Laboratory of Pediatrics, Chongqing, China

**Keywords:** neuroblastoma, ADRB2, clinical characteristics, prognosis, children

## Abstract

**Objective:**

Neuroblastoma (NB), originating from sympathetic spinal tissue, is a serious threat to the life of children. Especially in the high-risk group, an overall five-year survival rate less than 50% indicates an extremely poor clinical outcome. Here, the expression the of *β*-2 adrenergic (ADRB2) receptor gene in tumor tissues of children with NB was detected and the correlation between its expression and clinical characteristics and prognosis was analyzed.

**Methods:**

Forty-five tumor tissue samples and forty-eight paraffin sections of NB were obtained from Children’s Hospital of Chongqing Medical University from 2015 to 2021. Real-time fluorescence quantitative polymerase chain reaction (RT–qPCR) was utilized to detect the expression of ADRB2 at the mRNA level and immunohistochemistry (IHC) at the protein level.

**Results:**

For the RT–qPCR, the analysis showed that the expression of ADRB2 in the high-risk group was significantly lower (*P *= 0.0003); in addition, there were also statistically significant differences in Shimada classification (*P *= 0.0025) and N-MYC amplification (*P *= 0.0011). Survival prognosis analysis showed that the prognosis was better with high ADRB2 expression (*P *= 0.0125), and the ROC curve showed that ADRB2 has a certain accuracy in predicting prognosis (AUC = 0.707, CI: 0.530–0.884). Moreover, the expression of ADRB2, N-MYC amplification and bone marrow metastasis were the factors that independently affected prognosis, and at the protein level, the results showed that the differential expression of ADRB2 was conspicuous in risk (*P *= 0.0041), Shimada classification (*P *= 0.0220) and N-MYC amplification (*P *= 0.0166). In addition, Kaplan–Meier curves showed that the prognosis in the group with high expression of ADRB2 was better (*P *= 0.0287), and the ROC curve showed that the score of ADRB2 had poor accuracy in predicting prognosis (AUC = 0.662, CI: 0.505–0.820).

**Conclusion:**

ADRB2 is a protective potential biomarker and is expected to become a new prognostic biomolecular marker of NB.

## Introduction

Neuroblastoma (NB) is one of the most common extracranial malignant solid tumors in children, accounts for approximately 6%–10% of children’s neoplastic diseases (1) and 15% of tumor-related mortality (2, 3). Originating from the neural crest tissue of the sympathetic nervous system, NB mostly occurs in the retroperitoneal adrenal region (4). The special site of onset makes it difficult to be observed in the early stage; therefore, when patients come to the hospital due to clinical symptoms, the stage generally came advances, and the tumor erodes the surrounding blood vessels, tissues, and metastases, resulting in high mortality and poor outcomes (5–10). According to the Children’s Oncology Group (COG) (11), NB can be divided into a high-risk group, intermediate-risk group and low-risk group, and the latter two groups have been classified as non-high-risk groups because of their relatively good prognosis. However, in the high-risk group, multitherapy with surgery, chemoradiotherapy, and immunological therapies could alleviate the disease to a certain extent and increase the survival rate, and the overall survival rate is still not optimistic (12,13).

In the early stage, we downloaded the corresponding information from the Gene Expression Omnibus (GEO) database, identified and analyzed the differentially expressed genes in children with NB in the high-risk group and non-high-risk group by comprehensive bioinformatics, and finally determined that the *β*-2 adrenergic (ADRB2) receptor gene is a potential molecular marker that is related to the good prognosis of NB (14).

The ADRB2 gene is a protein-coding gene that is located at 5q31–32 on human chromosome 5 in a length of approximately 1.8 kb (15) and has been reported in various tumors (16–19). Research shows that ADRB2 receptor is a sevenfold-fold transmembrane G protein-coupled receptor and can be activated by *β*-receptor agonists, leading to the activation of guanine nucleotide-binding protein, and stimulating the production of cyclic AMP (cAMP). Subsequently, the increasing cAMP activates cAMP-directly-activated protein kinase A (PKA), protein kinase C (PKC) or exchange protein (EPAC), and activates additional signal transduction pathways to further regulate cell survival, movement, and proliferation (20–23). However, there are few studies on the ADRB2 in NB. Therefore, this study aims to explore the relationship between ADRB2 expression on clinical characteristics and the prognosis of NB by analyzing the expression level of the ADRB2 in tumor tissues, and provides a new molecular marker for NB.

## Data and methods

### General information

Forty-five children with NB who underwent surgical treatment in the Department of Oncology, Children’s Hospital of Chongqing Medical University from 2015 to 2021 were enrolled. The removed tumor tissues of children with NB were collected immediately and stored in a −80°C refrigerator. Inclusion criteria: (1) Diagnosed as primary NB; (2) Confirmed by pathology; (3) No treatment before operation. Exclusion criteria: (1) preoperative chemotherapy, radiotherapy, and other treatment; (2) Combined with other malignant tumors. In addition, 48 paraffin sections were obtained from the Pathology Department, Children’s Hospital of Chongqing Medical University, which were collected and made at the first operation. Moreover, a total of 36 fresh tissues and paraffin sections were from the same patient according to our statistics. All the patients enrolled were followed up regularly by outpatient service or telephone, and the time was up to December 31, 2021. The postoperative survival rate was calculated according to the follow-up results. This study was approved by the ethics committee of the Children’s Hospital of Chongqing Medical University. The parents whose children were enrolled provided an informed consent form.

### Real-time fluorescence quantitative polymerase chain reaction (RT–qPCR)

Total RNA was extracted from tumor tissue samples and reverse transcribed, then conducted the RT-qPCR experiments according to the manufacturer’s instructions. The results were calculated by the 2^^−ΔΔCt^ method and the primer sequences are shown in Table 1.

**Table 1:** The primer sequence.

Gene Name	Primer sequence	Length of production (bp)
ADRB2	5′- GGGTCTTTCAGGAGGCCAAA -3′	169
	5′- ATGCCTAACGTCTTGAGGG -3′	169
*β*-ACTIN	5′- AGTTGCGTTACACCCTTTCTT-3′	146
	5′- CACCTTCACCGTTCCAGTTTT-3′	146

### Immunohistochemistry (IHC)

After baking, dewaxing, hydration, and antigen repair, the paraffin sections were subjected to experimental operations according to the instructions of the immunohistochemistry kit provided by the reagent vendor, and finally sealed and observed under a microscope after natural drying. The judgment criteria were as follows: (1) Dyeing intensity: Cells are not colored, 0 points; pale yellow, 1 point; medium yellow or brown, 2 points; Dark brown or tan, 3 points; (2) Positive cell ratio: No positive cells, 0 points; less than 25%, 1 point; less than 50%, 2 points; less than 75%, 3 points; greater than 75%, 4 points. Finally, the score of the two parts were added: 0 to 3 points: negative expression; 4 to 7 points: positive expression.

### Statistical analysis

The results were analyzed by SPSS 25.0 software and GraphPad Prism 8.0. For the results of RT–qPCR, a nonpaired t test was performed for the comparison between the groups. According to the order of ADRB2 expression level, the low expression group and high expression group of ADRB2 were divided by the P50 boundary. The Kaplan‒Meier method was used to draw the overall survival curve and Cox regression analysis was used to analyze the independent influencing factors. For the results of IHC, each section was assessed as a score according to the judgment criteria to determine whether it was positively expressed and divided into the group of high and non-high expression due to the P50 boundary of the score. Next, the chi-square test was used to analyze the difference in groups, and Kaplan‒Meier curves were utilized to analyze the prognosis. A *P* value (*P*) < 0.05 was considered to indicate statistical significance.

## Results

### Clinical characteristics

This study enrolled 45 samples for RT–qPCR and 48 samples for IHC.

#### Characteristic of clinical samples for RT-qPCR

In the 45 samples used for RT**‒**qPCR, the statistics revealed that the median age was 840 days. In addition, 30 samples were in the high-risk group and 15 were in the non-high-risk group, 30 males and 15 females, 13 with the FH type of Shimada and 32 with the uFH type, 10 samples with N-MYC amplification and 35 with non-amplification.

#### Characteristic of clinical samples for IHC

For the IHC samples, the median age was 795 days. Moreover, there were 36 samples in the high-risk group and 12 in the non-high-risk group, 31 males and 17 females, 12 with the FH type of Shimada and 36 with the uFH type, and 16 samples with N-MYC amplification and 32 with non-amplification.

#### Characteristic of clinical samples from the same source

A total of 36 samples were from the same source, the median age was 765 days. In addition, 27 samples were in the high-risk group and 9 were in the non-high-risk group, 25 males and 11 females, 7 with the FH type of Shimada and 29 with the uFH type, 9 samples with N-MYC amplification and 27 with non-amplification.

### The consequence of RT–qPCR

#### Expression of ADRB2 in the total sample

**Figure 1:**
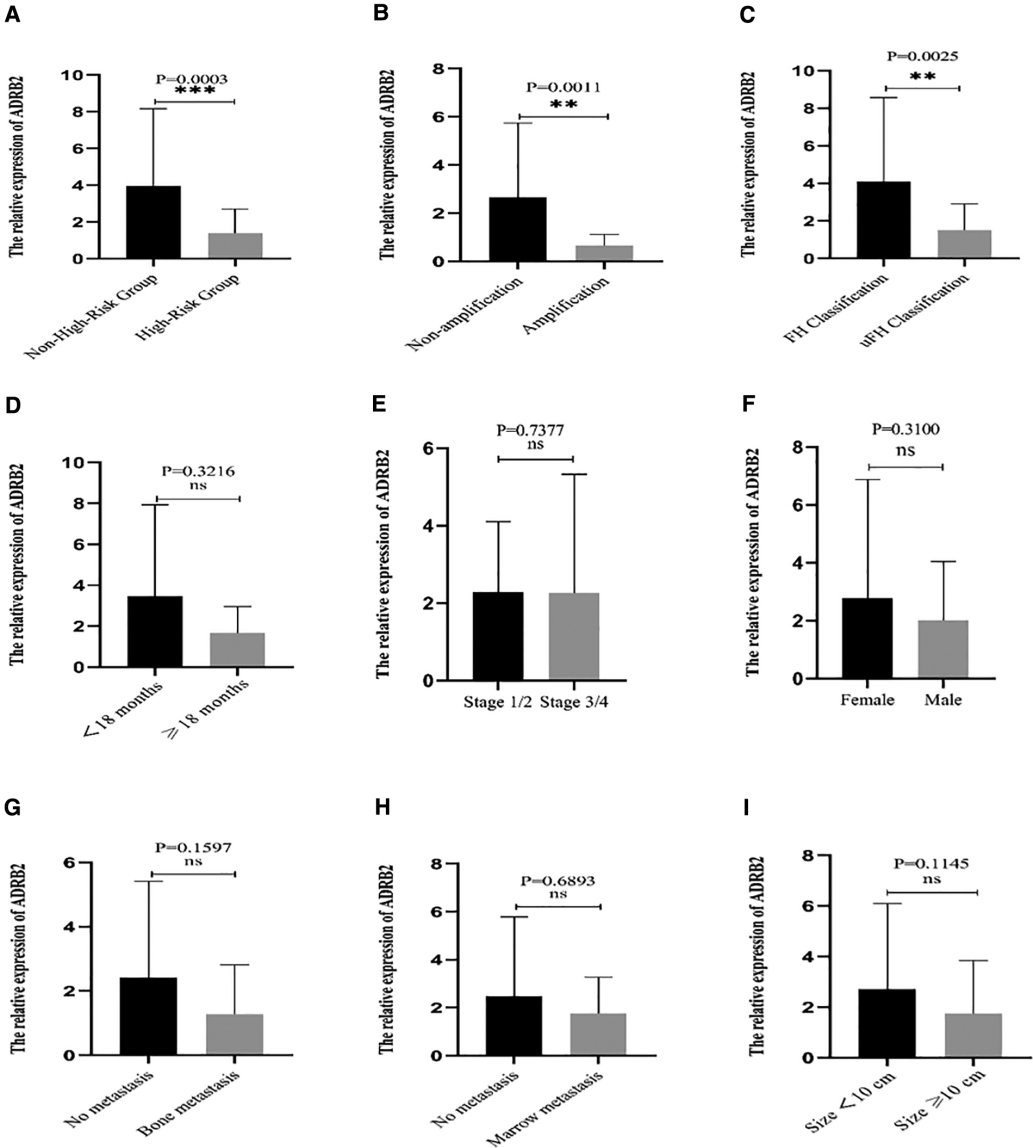
The correlation between the relative expression of ADRB2 and clinical features. (**A**) The expression of ADRB2 in the different risk groups; (**B**) The expression of ADRB2 in the different group of N-MYC amplification or not; (**C**) The expression of ADRB2 in the different Shimada classification; (**D**) The expression of ADRB2 in the different group of age less than 18 months or not; (**E**) The expression of ADRB2 in the different INSS stage; (**F**) The expression of ADRB2 in different gender; (**G**) The expression of ADRB2 in group of whether bone metastasis or not; (**H**) The expression of ADRB2 in the different group of whether bone marrow metastasis or not; (**I**) The expression of ADRB2 in the different group of whether tumor size greater than 10 cm or not.

The results showed that the relative expression level of ADRB2 in the high-risk group was significantly lower than that in the non-high-risk group (Figure 1A). The correlation between the expression of ADRB2 and clinical features showed that the expression of ADRB2 was higher in groups with the FH type (Figure 1C) and N-MYC non-amplification (Figure 1B), and more details are shown in Figure 1 and Table 2.

**Table 2:** The effect of ADRB2 gene expression and clinical features.

Parameters	Groups (*n*)	Expression $\left(\bar{X}\pm \mathrm{SD}\right)$	*P* value
Gender	Male (30)	2.0064 ± 2.0352	0.3100
	Female (15)	2.7707 ± 4.1073	
Age	<18 months (15)	3.4539 ± 4.4841	0.3216
	≥18 months (30)	1.6648 ± 1.2950	
N-MYC	Amplification (9)	0.6481 ± 0.4748	0.0011^a^
	Non-amplification (36)	2.6645 ± 3.0756	
Risk	High risk (30)	1.4038 ± 1.3094	0.0003^a^
	Non-high risk (15)	3.9761 ± 4.1940	
Stage	Stage 3/4 (37)	2.2572 ± 3.0681	0.7377
	Stage 1/2 (8)	2.2798 ± 1.8232	
Tumor Size	<10 cm (24)	2.7126 ± 3.3871	0.1145
	≥10 cm (21)	1.7453 ± 2.0953	
Shimada Type	FH (13)	4.1002 ± 4.1940	0.0025^a^
	uFH (32)	1.5141 ± 1.3965	
Marrow metastasis	Yes (14)	1.7681 ± 1.5037	0.6893
	No (31)	2.4839 ± 3.3056	
Bone metastasis	Yes (6)	1.2821 ± 1.5372	0.1597
	No (39)	2.4118 ± 3.0074	

^a^ Indicates a statistically significant difference.

#### Prognosis analysis of ADRB2 in the total sample

**Figure 2:**
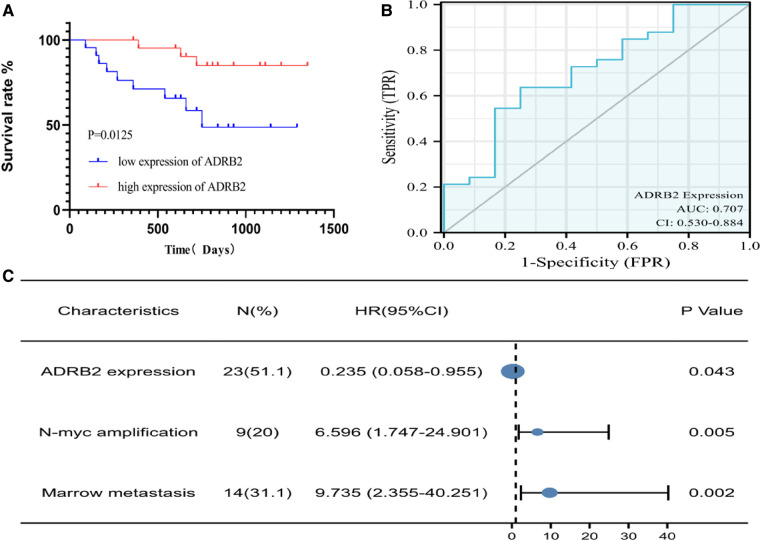
The Kaplan-Meier and ROC curve of ADRB2 and Forest plot of the independent factors. (A) The Kaplan-Meier curve for the prognosis of children with NB, the survival rate in group of high expression of ADRB2 is higher than that in the low expression group; (**B**) The ROC curve to analyze the prognosis predict ability of ADRB2 in children with NB, the results showed that the prognosis predictive ability of ADRB2 is a certain degree of accuracy; (**C**) The Forest plot of Multivariate Cox regression curve, the results showed that high expression of ADRB2, N-MYC amplification and bone marrow metastasis are the independent factors what affect the prognosis of children with NB.

According to the order of ADRB2 expression level, the low expression group and high expression group of ADRB2 were divided by the P50 boundary, and the Kaplan‒Meier curve suggested that the low expression group had a poor prognosis in NB patients (Figure 2A). In addition, the expression of ADRB2 was used to perform receiver operating characteristic (ROC) curve analysis to detect the feasibility of ADRB2 as a predictor of prognosis in children with NB, and the results showed that the predictive ability of ADRB2 has a certain accuracy (AUC = 0.707, CI = 0.530–0.884, Figure 2B).

Finally, univariate Cox regression analysis was performed with the expression of ADRB2, sex, Shimada classification, N-MYC amplification, age, tumor size, bone metastasis and bone marrow metastasis as independent variables and prognosis (survival/death) as a dependent variable. Then parameters with a *P* value less than 0.1 in the univariate analysis were selected for the multivariate regression analysis, and the results indicated that the expression of ADRB2, N-MYC amplification and bone marrow metastasis were independent factors that affected the prognosis (Figure 2C and Table 3).

**Table 3:** The Cox of Univariate and multivariate regression analysis.

Characteristics	Total (N)	Univariate analysis		Multivariate analysis	
		Hazard ratio (95% CI)	*P* value	Hazard ratio (95% CI)	*P* value
ADRB2 Expression	45	0.218 (0.059–0.809)	0.023^a^	0.235 (0.058-0.955)	0.043^a^
Gender	45	1.510 (0.406–5.606)	0.538		
Shimada Classification	45	397,895,286.721 (0.000-Inf)	0.998		
MYCN amplification	45	5.113 (1.639–15.949)	0.005^a^	6.596 (1.747–24.901)	0.005^a^
Bone Marrow metastasis	45	4.122 (1.302–13.053)	0.016^a^	9.735 (2.355–40.251)	0.002^a^
Bone metastasis	45	1.912 (0.413–8.841)	0.407		
Tumor size ≥10 cm	45	1.022 (0.329–3.172)	0.970		
Age ≥ 18 months	45	0.987 (0.296–3.286)	0.983		

^a^ Indicates a statistically significant difference.

Overall, the analysis found that ADRB2 was highly expressed in the non-high-risk group and high expression of ADRB2 gene was associated with good prognosis at the level of mRNA.

### The consequence of IHC

IHC was performed to verify the protein expression level of ADRB2 in clinical NB tissues.

#### Expression of ADRB2 in the total sample

Accounting for the results, 4 samples were judged to have positive expression in the high-risk group and 6 in the non-high-risk group. The positive expression rate was 11.11% in the high-risk group and 50% in the other group, and the chi-square test showed that the expression difference between the two groups was conspicuous (*P *= 0.0041). In addition, the correlation between the positive expression of ADRB2 and clinical features was analyzed. The results showed that no significant difference was found among the protein expression of ADRB2 with sex, age, tumor size, INSS stage, marrow and bone metastasis, but the results indicated that the positive expression of ADRB2 was related to Shimada classification (*P *= 0.0220) and N-MYC non-amplification (*P *= 0.0166). All the details are shown in Table 4.

**Table 4:** The correlation between positive expression of ADRB2 and clinical features.

Parameters	Number	Expression of ADRB2		*χ* ^2^	*P*
		Positive	Negative		
Overall	48	10 (20.83%)	38 (79.17%)		
Age				0.034	0.8544
≥18 m	30	6 (20%)	24 (80%)		
<18 m	18	4 (22.22%)	14 (77.78%)		
Gender				3.337	0.0677
Male	31	4 (12.90%)	27 (87.10%)		
Female	17	6 (35.29%)	11 (64.71%)		
Tumor Size				0.970	0.3264
≥10 cm	21	3 (14.29%)	18 (85.71%)		
<10 cm	27	7 (25.93%)	20 (74.07%)		
Risk				8.253	0.0041^a^
High-risk	36	4 (11.1%)	32 (88.9%)		
Non-high risk	12	6 (50%)	6 (50%)		
INSS Stage				0.298	0.5854
3/4	41	8 (19.51%)	33 (80.49%)		
1/2	7	2 (28.57%)	5 (71.43%)		
Shimada Type				5.245	0.0220
FH	11	5 (45.45%)	6 (54.55%)		
uFH	37	5 (13.51%)	32 (86.49%)		
N-MYC Amplification				5.742	0.0166^a^
Yes	15	0 (0%)	15 (100%)		
No	33	10 (30.30%)	23 (69.70%)		
Marrow metastasis				0.201	0.6543
Yes	21	5 (23.81%)	16 (76.19%)		
No	27	5 (18.52%)	22 (81.48%)		
Bone metastasis				0.6348	0.4256
Yes	9	1 (11.11%)	8 (88.89%)		
No	39	9 (23.08%)	30 (76.92%)		

^a^ Indicates a statistically significant difference.

#### Prognosis analysis of ADRB2 in the total sample

**Figure 3:**
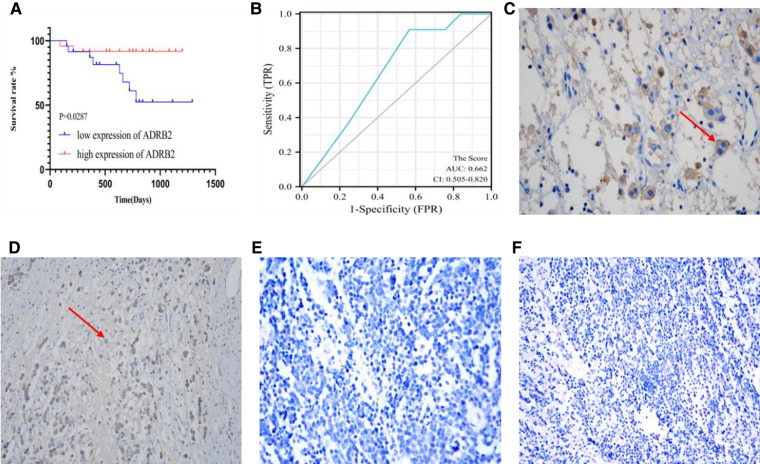
The image of IHC for Kaplan-Meier curve, ROC curve, and both positive (**C,D**) and negative (**E,F**) expression. (**A**) The Kaplan-Meier curve for the prognosis of children with NB, the prognosis in group of high expression of ADRB2 is better than that in the low expression group; (**B**) the ROC curve of IHC scores and prognosis shows a poor predictive ability; (**C**) This section belongs to INSS Stage 3, FH type and non-high-risk group, at the view of 400× field of IHC, the red arrow points to the positively expressed cells; (**D**) This section belongs to INSS Stage 2, uFH type and non-high-risk group, at the view of 200× of IHC, the red arrow points to the positively expressed cells; (**E**) This section belongs to INSS Stage 4, uFH type and high-risk group, at the view of 400× of IHC; (**F**) This section belongs to INSS Stage 3, uFH type and high-risk group, at the view of 200× of IHC.

According to the score order of each paraffin section, P50 was used as a cut-off to divided the patients into high expression group and low expression group. Kaplan‒Meier curve showed that high expression of ADRB2 was related to good prognosis (*P *= 0.0287, Figure 3A). In addition, the score was used to plot the ROC curve to predict the prognosis and the results showed that the IHC score had a poor predictive ability for prognosis (AUC = 0.662, CI = 0.505–0.820, Figure 3B). Finally, some of the IHC result images are displayed in Figure 3.

### Prognosis analysis of ADRB2 in the same source samples

As for the same source of fresh tissue and paraffin, according to the order of RT-qPCR expression values, the P50 value was used as a cut-off to divide the high and low expression groups. Based on this consequence, patients in IHC were divided into the two groups, and the chi square test indicated that the expression of ADRB2 were significantly (*P *= 0.0352). Besides, the analysis also showed that the Shimada classification (*P *= 0.0032) and N-MYC amplification status (*P *= 0.0013) were different between the two groups, all these expression tendencies was consistent with that in RT-qPCR.

In general, ADRB2 was highly expressed in the non-high-risk group and high expression of ADRB2 gene was associated with good prognosis at the level of protein.

## Discussion

With the continual improvement of molecular biology, people have gradually realized that molecular genetic variation plays an important role in cancer. Therefore, increasing number of studies on the molecular mechanism of NB have been conducted, and a considerable number of molecules have been considered to be related to the pathogenesis of NB, such as N-MYC, 1p, 11q, 17q and et al. (4, 24, 25). Although present molecular markers of NB could help to improve the prognosis in the high-risk group to a certain extent, it is still far from meeting people’s expectations as a long-term survival rate of less than 50% in the high-risk group (11, 12). Therefore, more biomolecule markers need to be discovered to improve the prognosis of children.

The MYC gene family, including MYC, N-MYC and L-MYC, has been confirmed to be highly expressed in various cancers (26). The transcriptional regulator N-myc is encoded by the MYCN gene, and has been confirmed to regulate many target genes involved in the cell cycle, apoptosis and DNA damage (27). In NB, N-MYC has been proven to have a good independent prediction ability of risk, and it is generally believed that N-MYC amplification means high risk and poor prognosis (1). According to our multivariate regression analysis, N-MYC amplification is an independent risk factor leading to poor prognosis, which was consistent with the current report. In addition, we found that samples that positively expressed ADRB2 were all in the N-MYC non-amplification group, suggesting that the expression of ADRB2 may be controlled by the N-MYC regulatory network. However, there are no relevant reference to confirm this opinion, so, this hypothesis needs further experimental verification.

Bone marrow metastasis is generally considered an adverse prognostic factor in NB, and it means that the patients need to undergo more therapeutic procedures. Some scholars have also studied the bone marrow metastasis in NB from the single-cell transcription perspective and found that metastatic tumor cells could reshape the bone marrow microenvironment (28). In addition, Fan (29) found that the prognosis of patients with bone marrow metastases was worse than those without bone marrow metastases. In our research, we also found that bone marrow metastasis was related to poor outcomes and was an independent risk factor for poor prognosis.

The ADRB2 is involved in the occurrence of a variety of tumors by affecting proliferation, invasion, immune regulation, angiogenesis and chemotherapy resistance (17, 30). Studies by some scholars have shown that using adrenaline to activate ADRB2 delays the apoptosis of cancer cells in prostate cancer, thus increasing the resistance to cytotoxic drugs, and proved that this anti-apoptotic effect is triggered by the ADRB2-PKA-MCL1 signaling pathway (18). It has also been reported that ADRB2 interferes with the autophagy of the Beclin1/VPS34/Atg14 complex in hepatocellular carcinoma, resulting in the reprogramming of glucose metabolism and the acquisition of sorafenib resistance (31). In our study, ADRB2 was highly expressed in the non-high-risk group, after compared with the expression of ADRB2 different clinical groups, we found that the ADRB2 was also highly expressed in the Shimada type FH and N-MYC non-amplification group. This suggests that high expression of this gene is associated with the groups which were clinically considered to have a good prognosis. In addition, the ROC curves also showed the prediction ability for prognosis of ADRB2 is valuable. Moreover, Kaplan‒Meier curves also suggested that the prognosis of children with high ADRB2 expression was generally better than those with low expression. Our results indicated that ADRB2 is a good prognosis-related gene in NB.

The deficiency of this study is that the sample size was relatively small, and molecular mechanism-related experiments were not performed. Therefore, a larger sample size and deep study of the mechanism of ADRB2 in NB are needed.

In conclusion, the expression of ADRB2 is higher in the non-high-risk group than that in the high-risk group, and the prognosis of children with high expression of ADRB2 is also better, which suggests that ADRB2 may hinder the progression of the tumor. ADRB2 may be a protective potential molecular marker in the occurrence and development of NB and is expected to become a prognostic biomarker.

## Data Availability

The original contributions presented in the study are included in the article/**Supplementary Material**, further inquiries can be directed to the corresponding author/s.
