# Supplementary material for: Expression of ADRB2 in children with neuroblastoma and its influence on prognosis
Source: Front Surg. 2022 Nov 2;9:1026156. doi: 10.3389/fsurg.2022.1026156 (PMC9666690; doi:10.3389/fsurg.2022.1026156)
Supplement: Supplementary file 1 [file Datasheet1.docx]

**Procedures of RT-qPCR**

The total RNA of NB tumor tissues was extracted by TRIzol reagent (Vazyme, China), the concentration and purity of the RNA sample were determined by Nano DropOne (Thermo Scientific), and qualified RNA samples were reverse transcribed into cDNA. Reverse transcription kits and RT‒qPCR kits were both purchased from ACCURATE, China. According to the instructions provided by the reagent manufacturer, RT‒qPCR was performed to detect the expression level of ADRB2 in tumor tissue samples. Reaction conditions: The volume of the reaction was 20 μl and the total number of cycles was 40. Denaturation at 95 ℃ for 30 s, annealing at 60 ℃ for 30 s and extension at 72 ℃ for 30 s were followed by pre-denaturation at 95 ℃ for 5 min.

**Procedures of IHC**

Paraffin sections were first baked in an oven at 60°C for 1 hour, fully dewaxed with xylene and rewatered with an ethanol gradient. Next the sections were placed in a beaker with citric acid repair solution (0.4 grams of citric acid and 3 grams of trisodium citrate were completely dissolved in 1 liter of double-distilled water, and the pH was adjusted to 6 with dilute hydrochloric acid), heated to boiling in a microwave oven and boiled for 20 minutes, after which the beaker was removed, cooled naturally to room temperature and washed with 10% phosphate‐buffered saline (PBS) 3 times. Afterward, according to the IHC kit (including Reagent 1, 2, 3, ZS-BIO, China) instructions provided by the reagent vendor, the paraffin sections were treated with endogenous peroxidase blocker (Reagent 1) for 10 minutes, and washed with 10% PBS again. After that, the sections were further blocked with 0.5% bovine serum albumin (BSA, Solarbio, China) at 37°C for 1 hour. Then, the primary antibody (Abcam, UK) against ADRB2 was added and diluted with BSA solution at a ratio of 1:100 in a 4°C refrigerator for 16 hours. The next day, the sections were washed with 10% PBS, incubated with a reaction enhancer (Reagent 2) for 20 minutes, washed, and incubated with the secondary antibody (Reagent 3) for 20 minutes. The secondary antibody was washed out with 10% PBS, and a DAB chromogenic Kit (ZS-BIO, China) was used for color rendering. Furthermore, the sections were dye with hematoxylin for 10 seconds, rinsed with running water for 5 minutes, removed with hydrochloric acid alcohol for 5 seconds, rinsed with running water for 5 minutes, reversed blue with saturated lithium carbonate for 5 seconds, rinsed with running water again for 5 minutes, dehydrated with gradient ethanol, soaked with xylene for 20 minutes and sealed. The sections were observed under a microscope after being dried naturally.
